# A nanoluciferase-tagged Schmallenberg virus (SBV): an efficient tool for measuring and tracking viral infection dynamics

**DOI:** 10.1099/jmm.0.002084

**Published:** 2025-10-17

**Authors:** Franziska Sick, Andrea Aebischer, Martin Beer, Kerstin Wernike

**Affiliations:** 1 Institute of Diagnostic Virology, Friedrich-Loeffler-Institut, Greifswald-Insel Riems, Germany

**Keywords:** high-throughput screening, nanoluciferase reporter, neutralization assay, reverse genetics, Schmallenberg virus

## Abstract

**Introduction.** Schmallenberg virus (SBV) is an arthropod-borne virus and belongs to the Simbu serogroup within the family *Peribunyaviridae*, genus *Orthobunyavirus*. Infection of naïve ruminants at critical stages of gestation can result in severe congenital malformations or abortion.

**Gap statement.** Tools to measure virus infection parameters in cell culture such as replication efficiency, as well as neutralization assays, are mainly available in the form of assays based on the evaluation of cytopathic effects. The methods are labour-intensive and low-throughput, as they require long incubation periods of several days. Tools such as tagged SBV that allow for fast and automated readout are missing.

**Aim.** We aimed to develop a tagged SBV that can be used for assays with fast and automated read-out.

**Methodology.** We report the construction of a recombinant SBV stably expressing the nanoluciferase (nluc) enzyme (rSBV_nluc). Using reverse genetics, the nluc gene was integrated into the genome of an SBV variant naturally harbouring a large deletion within the Gc-head domain. The nluc gene was inserted into this locus.

**Results.** The nluc-tagged virus showed *in vitro* no signs of attenuation and identical replication properties when compared to the parental virus in baby hamster kidney (BHK-21) cells. Our results demonstrate a new approach for rapid access to SBV replication. By performing nluc assays, we were able to track viral replication and also virus uptake in detail. We further evaluated neutralization properties of an SBV variant, which is lacking a major part of its antigenic domain (Gc-head) and developed a nanoluciferase activity-based serum neutralization assay.

**Conclusion.** Overall, the nluc-tagged SBV is a suitable tool that further facilitates the study of viral infection dynamics and allows for high-throughput assays.

## Introduction

Schmallenberg virus (SBV) is a member of the Simbu serogroup within the family *Peribunyaviridae*, genus *Orthobunyavirus*. After its first appearance in 2011, SBV is now enzootic in most European countries [1,2]. As an arthropod-borne virus, SBV is transmitted by *Culicoides* biting midges [3,4]. Acute infection causes a mild disease in ruminants. However, when pregnant ruminants are infected, congenital malformations or abortion may occur [1,5].

The enveloped SBV particles are spherical and 100 nm in diameter [6]. They comprise a single-stranded RNA genome of negative polarity. The genome is divided into three genome segments that encode only four structural and two non-structural proteins. The small (S) segment encodes for the nucleocapsid protein N and in an overlapping reading frame for the non-structural protein NSs. The medium (M) segment encodes for the glycoproteins Gn and Gc and for the non-structural protein NSm. The large (L) segment encodes for the RNA-dependent RNA polymerase [7].

Tools to measure virus infection parameters in cell culture such as replication efficiency or virus uptake and also neutralization assays are available in the form of assays based on cytopathic effect (CPE) and immunofluorescence staining of cells [6,8]. However, these methods have a low throughput as they are labour-intensive and take several days from test to result.

Recombinant viruses carrying reporter genes have been widely used to study various viruses [9,12]. Bioluminescence reporters such as luciferase enzymes are highly suitable for studying viral infections because of their high sensitivity. Nanoluciferase (nluc) is a 19 kDa luciferase enzyme. Engineered from the deep-sea shrimp, it produces bioluminescence of high intensity upon binding to its substrate, furimazine. Nluc has a ∼150-fold greater specific activity than firefly luciferase [13,14].

For SBV, a reverse genetics system (RGS) is established that enables the generation of genetically modified recombinant SBV (rSBV) [8]. However, the insertion of exogenous genes into the viral genome poses the risk of impairment of viral growth or genetic stability [15,16]. The crucial point for the successful rescue of a recombinant virus carrying a reporter gene is the identification of a suitable locus for the insertion of the reporter gene or finding viral fragments suitable for replacement [15]. rSBV with an exogenous gene insertion has not yet been developed.

The strain SBV_D281/12, isolated from a malformed foetus, naturally carries a large deletion of 555 nt in the hypervariable region [17,18] of the Gc-coding gene within the M segment [19]. In this study, we used this locus for the insertion of the nluc gene to successfully generate nluc-tagged rSBV. The rescued rSBV_D281/12_nluc behaves similarly to the parental virus in terms of growth kinetics, and nluc is stably expressed throughout virus passage. Therefore, this newly generated tagged virus is a suitable and sensitive tool to study SBV infections by providing easy detection and tracking capabilities.

## Methods

### Cells and viruses

BHK-21 [RIE164 (CCLV)] and BSR-T7/5 [RIE583 (CCLV)] cells were obtained from the Collection of Cell Lines in Veterinary Medicine at the Friedrich-Loeffler-Institut, Greifswald-Insel Riems, Germany. BHK-21 cells were grown in Minimum Essential Medium (MEM) supplemented with 10% fetal calf serum (FCS) at 37 °C and 5% CO_2_. BSR-T7/5 cells were cultivated in Glasgow modified Eagle medium supplemented with 10% FCS at 37 °C and 5% CO_2_.

rSBV_D281/12, based on wt SBV_D281/12, was previously described [19]. The nluc-tagged rSBV_D281/12_nluc was generated by reverse genetics, as described below. The viruses used in this study were propagated as a virus stock and titrated on BHK-21 cells. Virus titres were calculated as 50% tissue culture infective dose per ml (TCID_50_/ml) according to Spearman and Kärber [20,21]

### Plasmid construction and purification

All plasmids were generated by the restriction-free cloning method using Phusion PCR [22] and confirmed by Sanger sequencing. Cloning PCRs were carried out with Phusion HF (New England Biolabs, Frankfurt am Main, Germany), following the standard molecular biology procedures. Plasmid DNA was purified by using QIAGEN Plasmid Midi Kits (QIAGEN, Hilden, Germany) according to the manufacturer’s protocol.

cDNA of the S, M and L segments of virus isolates SBV_D281/12 was previously cloned into the pTRiboSM2- vector [23], which was kindly provided by F. Weber (Justus-Liebig-Universität Gießen).

**Fig. 1.**
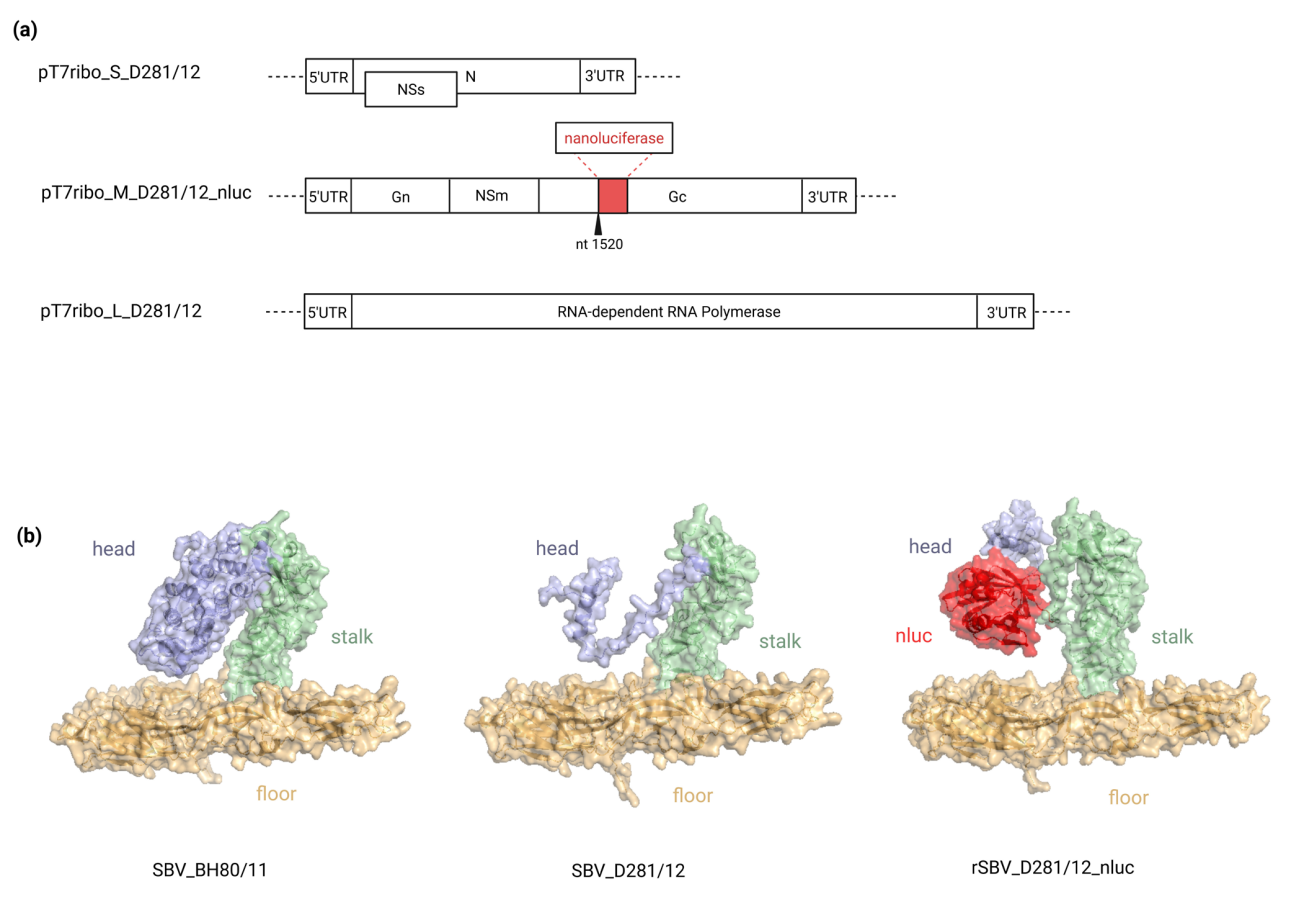
(a) Schematic depiction of the rescue plasmids for the generation of the nluc-expressing SBV. The pTRibo vector is shown as a dotted line. The nluc gene was inserted downstream of nt position 1520, within the coding region of the Gc glycoprotein. UTR, untranslated region. Figure created with biorender.com. (b) AlphaFold structure predictions of the monomeric Gc protein of wt SBV_BH80/11, isolate SBV_D281/12 and the rSBV_D281/12_nluc. The three different sections of the Gc ectodomain are coloured in light blue (head), green (stalk) and orange (floor). The nluc-enzyme inserted in the head domain of rSBV_D281/12_nluc is highlighted in red.

The plasmid pT7ribo_SBV_D281/12_M [19] served as the template for the construction of the plasmid pT7ribo_SBV_D281/12_M_nluc. In the M segment, the SBV isolate D281/12 has a deletion of the Gc-head domain, which was used as the locus for insertion of the nluc gene (Fig. 1a).

The nluc gene was inserted using phusion PCR techniques downstream of nt 1520 with a megaprimer, which was generated using the primers SBV_M_nluc_F (5′-CAA AAT AAA ACA AGC TCA CCA GTT ATG GTC TTC ACA CTC GAA GAT TTC-3′) and SBV_M_nluc_R (5′-GAG GGG TTT TTT CTA TTG TCA CGT TTG CCG CCA GAA TGC GTT CGC ACA G-3′). A DNA plasmid containing the nluc sequence served as the template for the megaprimer.

### Generation of rSBV_D281/12_nluc

For the recovery of rSBV, a previously published three-plasmid rescue system [8] was used with minor modifications. Shortly, 80% confluent layers of BSR-T7/5 cells were grown in six-well plates. Each well was transfected with 3 µg each of the recombinant S, M and L segment plasmids using Lipofectamine 3000 transfection reagent (Invitrogen, Thermo Fisher Scientific, Waltham, USA) according to the manufacturer’s protocol. Supernatants of transfected cells were harvested on days 4 to 5 post-transfection. Five hundred µl of supernatant were inoculated into 2.5 ml BHK-21 cell suspension and grown in six-well plates to screen for the presence of recombinant virus. Successful rescue of the recombinant virus was demonstrated by the observation of CPE. Rescued viruses were further passaged on BHK-21 cells, and virus stocks were generated from the third passage (P3). P3 was used for the described infection experiments unless stated otherwise. The correctness of gene sequences was verified by Sanger sequencing using the Big Dye Terminator Mix (Applied Biosystems, Darmstadt, Germany) of PCR fragments derived from viral RNA (Onestep RT-PCR Kit, QIAGEN, Hilden, Germany) as previously described [17].

### Virus titration and growth kinetics

*In vitro* growth kinetics were performed in 24-well plates using BHK-21 cells. The cells were inoculated with rSBV_D281/12 (P3) and rSBV_D281/12_nluc (P3 and P8) at a multiplicity of infection (MOI) of 0.1. Virus adsorption lasted for 1 h at 37 °C. Subsequently, cells were washed with PBS once, and 1 ml of fresh cell culture medium was applied to the cells. Supernatants were collected at 0 (before virus adsorption), 8, 24, 48 and 72 h post-infection (hpi) and were titrated on BHK-21 cells. Titres are displayed as TCID_50_/ml. Statistical analysis of the growth kinetics was done by performing a two-way ANOVA test using GraphPad Prism 10.4.0 software to analyse viral growth of rSBV_D281/12 (P3) and rSBV_D281/12_nluc (P3) and rSBV_D281/12_nluc (P8) over the course of time. Effects were regarded as significant when *P*<0.05.

### Nanoluciferase assay and adsorption testing

BHK-21 cells were seeded in 96-well plates one day prior to the experiment. Cells were inoculated with rSBV_D281/12_nluc at an MOI of 0.1. Virus adsorption lasted from 0 to 60 min in the virus adsorption test; in the other nluc tests, it lasted for 10 min. Subsequently, cells were washed with PBS and 100 µl of cell culture medium containing 5% FCS was added. For the measurement of the nluc activity, the Nano-Glo luciferase assay (Promega, Madison, USA) was performed according to the manufacturer’s protocol at different time points. Briefly, 100 µl of assay buffer and substrate (ratio 50 buffer:1 substrate) were added to the sample in the 96-well cell culture plate. After 3 min of incubation at room temperature (lysis of the cells), the mixture was transferred to a black 96-well plate (Perkin Elmer, Waltham, USA). The relative light units (RLU) were recorded by a Tecan Infinite F200 Pro Reader (Tecan, Männedorf, Switzerland) with an integration time of 2,000 ms. In the kinetic, the time points − 10 min (add virus, withdraw immediately), 0 min (after 10 min of virus adsorption), 1 h, 2 h, 3 h, 4 h, 5 h, 6 h, 7 h, 8 h, 16 h, 20 h and 24 h post-infection were measured.

### Stability of the integrated nanoluciferase gene

To test the stability of rSBV_D281/12_nluc in cells, the virus was serially passaged on BHK-21 cells until the eighth passage by passaging 20 µl of supernatant to the subsequent passage in a T25 cell culture flask containing 10 ml cell culture medium. The luciferase activity of P3 and P8 was evaluated in an nluc assay at 0 hpi and 24 hpi and compared to the parental virus rSBV_D281/12. Moreover, the nluc gene, together with its flanking sequences of the eighth passage, was Sanger sequenced as described above.

### Alphafold structure predictions

Models were generated with AlphaFold 3 and the AlphaFold Server [24] using the Gc sequences of the SBV strains SBV_BH80/11 (representing SBV with full-length Gc, GenBank accession number M segment: HE649913), SBV_D281/12 (GenBank accession number M segment: PP616750) and the Gc sequence of SBV_D281/12_nluc. Images were edited using the PyMol visualization software (Schrödinger LLC, New York, USA).

### Nanoluciferase activity-based serum neutralization assay (SBV nluc-SNT)

A conventional serum neutralization test (SNT) against SBV [25] was adapted to analyse sera via nluc assays. An SBV-positive reference serum, obtained from experimentally infected cattle [25], was compared to an SBV-negative control serum. Subsequently, a set of ten serum samples (cattle and sheep) from an international proficiency trial of SBV diagnostics was analysed [26]. For the SBV nluc-SNT, the serum samples were diluted in MEM in a 1/5 ratio and titrated in twofold dilutions until 1/320. Fifty µl of the diluted sera were incubated with 50 µl of MEM containing ~100 TCID_50_ of rSBV_D281/12_nluc per well in 96-well microtiter plates for 2 h at 37 °C. Virus incubated without any serum served as a virus-positive control. Subsequently, the serum dilution/virus mixtures were then added to BHK-21 cells that were seeded in 96-well plates 1 day before the experiment. The nluc assay was performed immediately at 0 hpi to detect the level of background signals and at 24 hpi. Therefore, one plate was prepared for each time point. Samples were tested in duplicate on each plate. The result is interpreted by comparing the 0 hpi and the 24 hpi value. Neutralization is detected when no rise in RLU from 0 to 24 hpi is observed. When RLU is higher at the 24 hpi measurement, no neutralization is detected.

## Results

### Construction of SBV nluc expression plasmids and virus rescue

For the construction of the rescue plasmid pT7ribo_SBV_D281/12_M_nluc, the plasmid pT7ribo_SBV_ D281/12_M [19] served as a template. The nluc gene was successfully inserted after the nt 1520 in the coding region of the Gc protein, a region where a natural deletion of 555 nt occurred in the wt SBV_ D281/12 compared to circulating SBV strains from viremic ruminants.

For virus rescue of rSBV_D281/12_nluc, the rescue plasmid pT7ribo_SBV_D281/12_M_nluc was transfected together with pT7ribo_SBV_D281/12_S and pT7ribo_SBV_D281/12_L into BSR-T7/5 cells. After three passages of the supernatants, the complete viral genome of rSBV_D281/12_nluc was sequenced and compared to the cDNA plasmid used for virus rescue. All sequences were correct; however, in the M segment, a single point mutation at nt 3001 (A to C) occurred for unknown reasons.

### Virus characterization on BHK-21 cells and stability tests

**Fig. 2.**
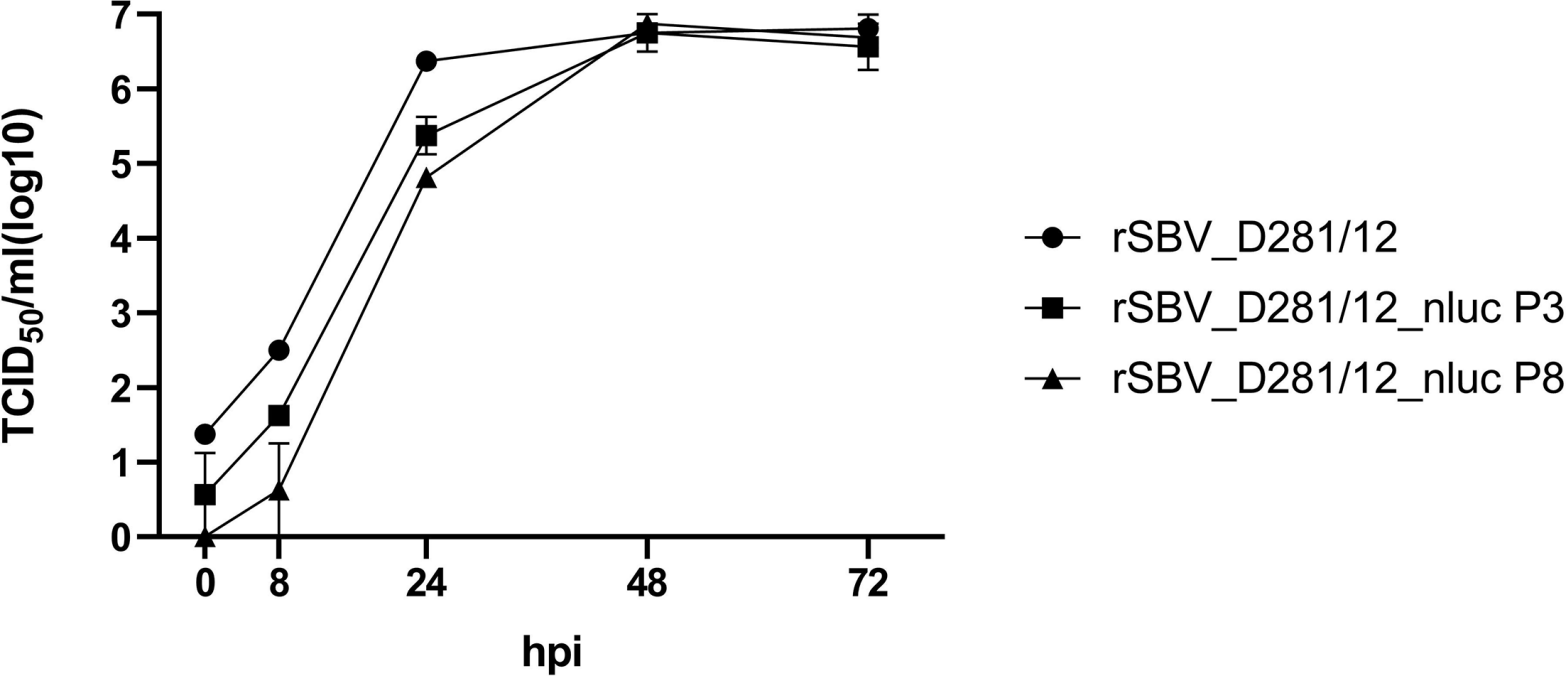
Growth kinetics of rSBV_D281/12, rSBV_D281/12_nluc P3 and P8 using BHK-21 cells. Cells were infected with MOI 0.1. The supernatants were harvested at 0, 8, 24, 48 and 72 hpi, and the virus titre was determined by virus titration on BHK-21 cells. Kinetics were performed in two biological replicates and for each time point, virus titration was performed in eightfold replicates. The dots of the graphs symbolize the mean values. Error bars indicate the mean±sd between the biological replicates.

**Fig. 3.**
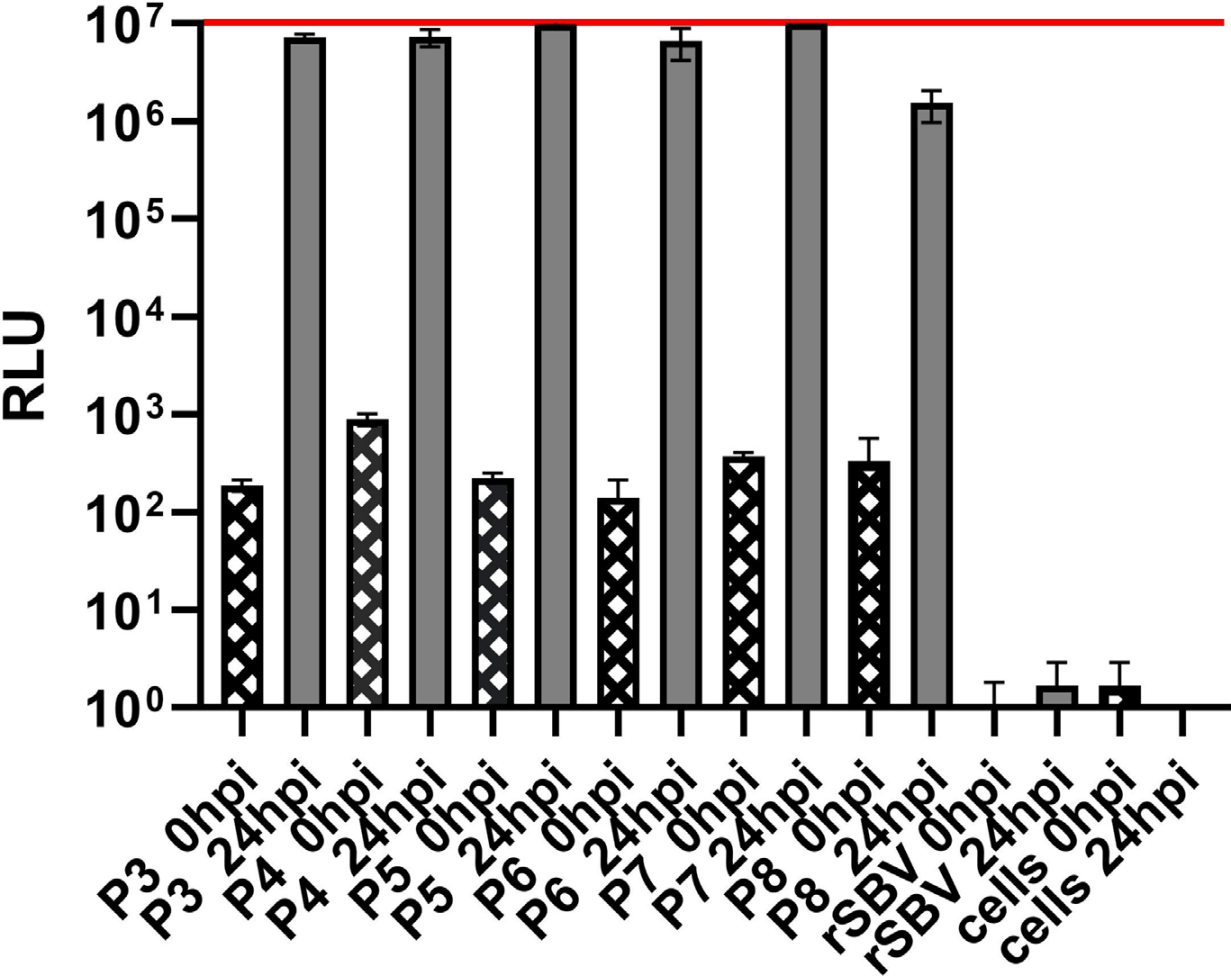
Nanoluciferase activity of lysed BHK-21 cells infected with rSBV_D281/12_nluc (passage P3 to P8) and the parental virus rSBV_D281/12 (rSBV) lacking the nluc and uninfected cells was compared. Assays were performed at 0 and 24 hpi in triplicate for each value, and RLU were measured. The columns symbolize the mean value. The error bars indicate the mean±sd. The red line indicates the maximal RLU that the Tecan reader can measure (10^7^). P3 throughout to P8 of rSBV_D281/12_nluc produced similar levels of bioluminescence signal at 0 and 24 hpi, whereas the parental virus and the uninfected control showed a low background signal only.

rSBV_D281/12 was compared to rSBV_D281/12_nluc in a growth kinetics study on BHK-21 cells (Fig. 2). Both viruses showed similar growth characteristics, with final titres of 10^6^ to 10^7^ TCID_50_/ml at 72 hpi. No difference in growth behaviour could be observed between passages three and eight of rSBV_D281/12_nluc. Two-way ANOVA analysis of the growth kinetics revealed that there was no statistically significant interaction between the effects of virus strain/virus passage and time (*P*=0.1105). The virus strain/virus passage revealed no statistically significant effect on viral growth (*P*=0.2352). However, the analysis showed that the factor time did have a statistically significant effect on viral growth (*P*=0.0049). The nluc sequence and flanking genomic regions of P8 were sequenced and compared to P3. No differences in the sequence were observed (data not shown). Nluc activity of P3 to P8 measured at 0 hpi and 24 hpi was comparable and showed an increase of five log10 steps at 24 hpi compared to 0 hpi. The parental virus rSBV_D281/12 without the nluc and the uninfected controls showed a low background signal only (Fig. 3). The results indicate that the insertion of the nluc gene did not affect viral replication *in vitro* and the recombinant SBV-nluc remained stable throughout the serial passages.

### Kinetics of nanoluciferase activity

**Fig. 4.**
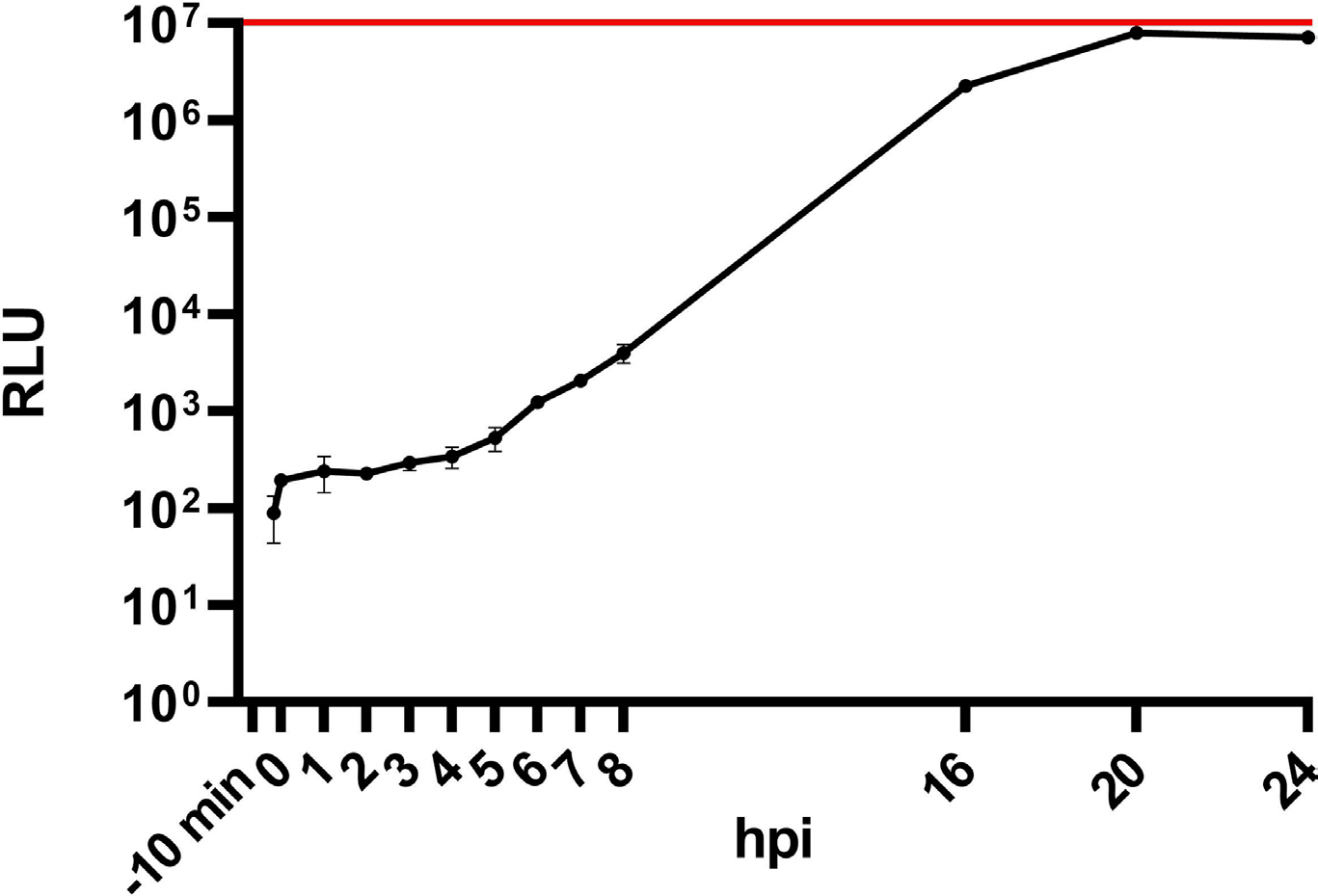
Nanoluciferase activity kinetics of lysed BHK-21 cells infected with rSBV_D281/12_nluc. Assays were performed at −10 min, 0 min (after 10 min of virus adsorption), 1 h, 2 h, 3 h, 4 h, 5 h, 6 h, 7 h, 8 h, 16 h, 20 h and 24 h post-infection in three replicates for each value, and RLU was measured. The dots of the graphs symbolize the mean values. The error bars indicate the mean±sd. The red line indicates the maximal RLU that the Tecan reader can measure (10^7^). An increase in RLU signals is detected from 6 hpi onwards and reaches its peak at 20 hpi.

Nluc assays of rSBV_D281/12_nluc infected BHK-21 cells were performed as a kinetic assay at different time points after infection (Fig. 4). From 0 to 5 hpi, the signal remained in a range of 2×10^2^ to 5×10^2^ RLU. From 6 to 8 hpi, a first increase of the RLU signal was detectable (1.2×10^3^ to 4×10^3^ RLU). At 16 hpi, the RLU signal increased to 2.3×10^6^. It reached its peak of 8×10^6^ RLU at 20 hpi and remained at this level until the endpoint of the kinetic at 24 hpi (Fig. 4).

### Virus uptake Nluc assay

**Fig. 5.**
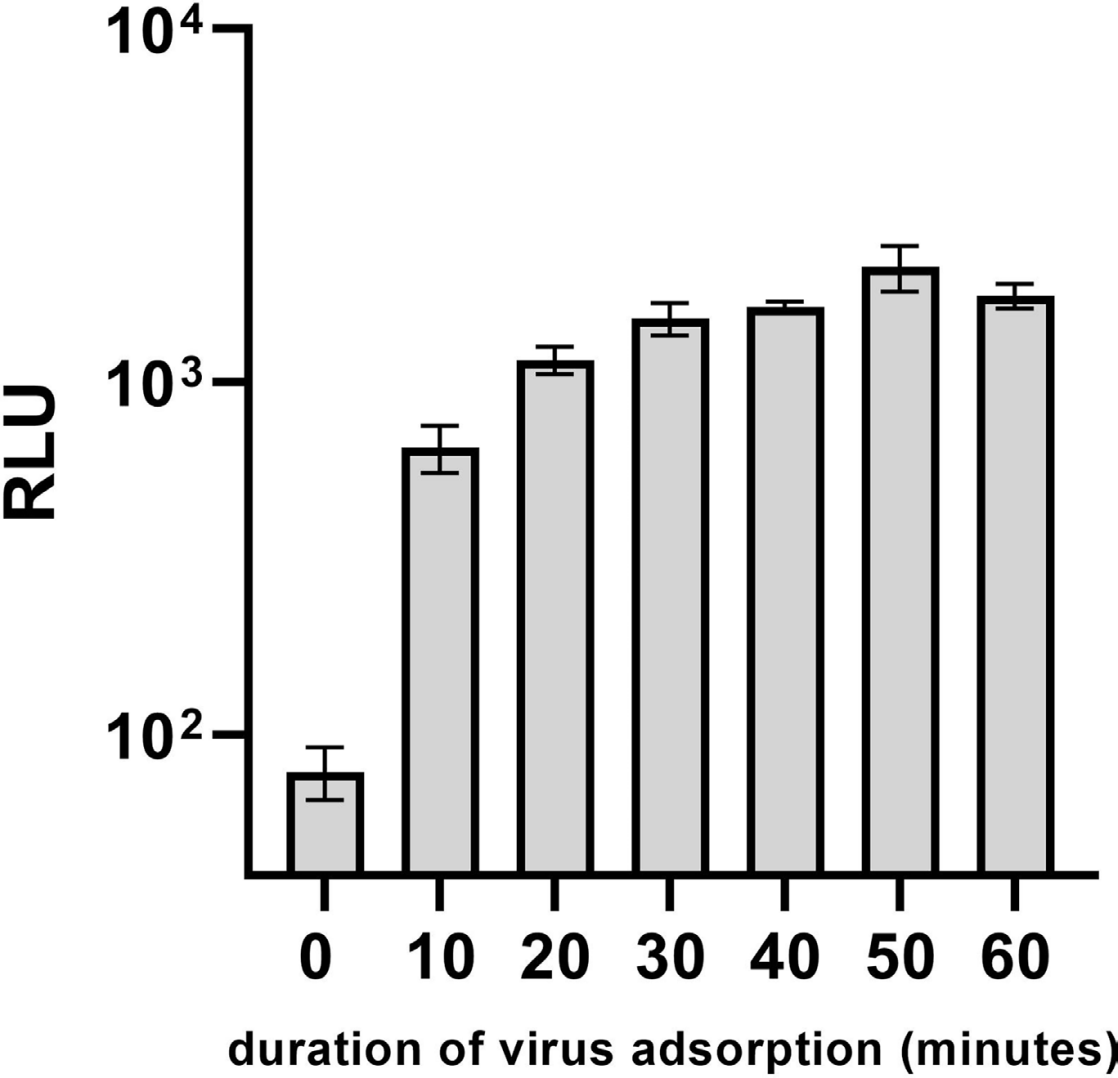
Nanoluciferase activity of lysed BHK-21 cells infected with rSBV_D281/12_nluc P3. The time of virus adsorption was compared and lasted from 0 to 60 min. After adsorption, the virus inoculum was removed, the cells were washed with PBS, and fresh medium was added to the cells. Subsequently, an nluc assay was performed, and the RLU was measured. Assays were performed in triplicate. The columns symbolize the mean value. The error bars indicate the mean±sd. A tenfold rise in RLU signal could be detected for an adsorption duration of only 10 min.

To investigate the speed of virus uptake, we incubated BHK-21 cells for different durations (0, 10, 20, 30, 40, 50 and 60 min) with rSBV_D281/12_nluc, removed the inoculum, washed the cells and directly performed a nluc assay. The signal for cells inoculated for 0 min adsorption time is below 10^2^ (7.9×10^1^ RLU) and can be considered as the background signal. After 10 min of virus adsorption, the RLU signal of the nluc assay was approximately tenfold above the background (6.5×10^2^ RLU), which did not rise much higher after 20 to 60 min (1×10^3^ to 2×10^3^) (Fig. 5).

### AlphaFold modelling of the Gc-nluc protein

In order to investigate the structure and position of the Gc-nluc construct in the context of the full-length protein, AlphaFold models were generated. By comparing the predictions for the rSBV-nluc virus, isolate SBV_D281/12 and SBV_BH80/11 (full-length protein), it could be demonstrated that the insertion of the nluc enzyme in the head domain of D281/12 has no impact on the conformation of the Gc ectodomain (Fig. 1b).

### Nanoluciferase activity-based serum neutralization assay (SBV nluc-SNT)

**Fig. 6.**
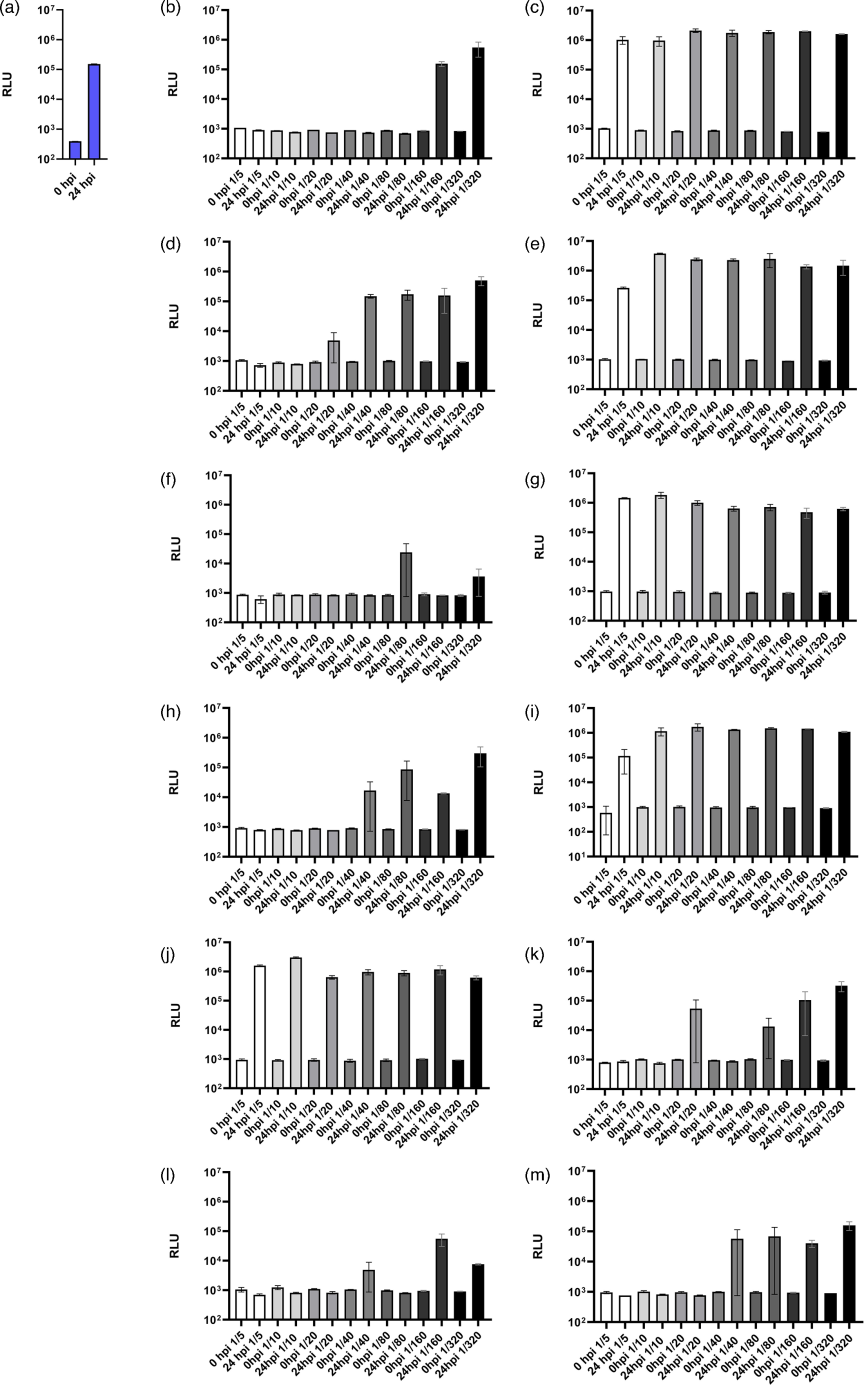
Nanoluciferase activity-based serum neutralization assay. (a) virus control with no serum, (b) SBV-positive control (neutralizing titre 1/80), (c) negative control (negative), (d) SBV-S1 (1/10), (e) SBV-S-2 (negative), (f) SBV-S-3 (1/40), (g) SBV-S-4 (negative), (h) SBV-S-5 (1/20), (i) SBV-S-6 (negative), (j) SBV-S-7 (negative), (k) SBV-S-8 (1/10), (l) SBV-S-9 (1/20), (m) SBV-S-10 (1/20). Assays were performed in two replicates. The columns symbolize the mean value. The error bars indicate the mean±sd.

To investigate the neutralization properties of a virus that is lacking a major part of its antigenic domain and to test the suitability of the nluc-tagged virus for application in a serum neutralization assay, two sera from cattle with known SBV antibody status, one positive and one negative, were evaluated. The SBV-positive serum had a titre of 1/240 ND_50_ in the conventional SNT. In the nluc assay, neutralization was detected for the SBV-positive reference serum until a dilution of 1/80. In the subsequent dilutions, no neutralization was detected. No neutralization was detected for the SBV negative control serum (Table 1, Fig. 6). Subsequently, the newly established SBV nluc-SNT was applied to the evaluation of a set of ten serum samples from an international proficiency trial for SBV serology. All negative samples SBV-S-2, SBV-S-4, SBV-S-6 and SBV-S-7 were correctly identified as not neutralizing. All positive samples (SBV-S-1, SBV-S-3, SBV-S-5, SBV-S-8, SBV-S-9 and SBV-S-10) were correctly identified as SBV-seropositive (Table 1, Fig. 6). However, the neutralizing titres of the seropositive samples were lower in the SBV nluc-SNT than in the conventional SNT. For example, the sample SBV-S-1 had a neutralizing dose of 50% of 1/28 in the conventional SNT and a neutralizing dose of 1/10 in the nluc-SNT. A comparison of the results of the conventional SNT and the nluc-SNT is provided in Table 1.

**Table 1.** Comparison of the SBV antibody status of tested samples in the conventional SNT (unit: neutralizing doses 50%) and nluc-SNT (unit: neutralizing dose)

Serum sample	Conventional SNT [25,26]	nluc-SNT
SBV positive control	SBV antibody + (1/240)	SBV antibody + (1/80)
SBV negative control	SBV antibody − (< 1/5)	SBV antibody − (< 1/5)
SBV-S-1	SBV antibody + (1/28)	SBV antibody + (1/10)
SBV-S-2	SBV antibody − (< 1/5)	SBV antibody − (< 1/5)
SBV-S-3	SBV antibody + (1/90)	SBV antibody + (1/40)
SBV-S-4	SBV antibody − (< 1/5)	SBV antibody − (< 1/5)
SBV-S-5	SBV antibody + (1/28)	SBV antibody + (1/20)
SBV-S-6	SBV antibody − (< 1/5)	SBV antibody − (< 1/5)
SBV-S-7	SBV antibody − (< 1/5)	SBV antibody − (< 1/5)
SBV-S-8	SBV antibody + (1/57)	SBV antibody + (1/10)
SBV-S-9	SBV antibody + (1/22)	SBV antibody + (1/20)
SBV-S-10	SBV antibody + (1/71)	SBV antibody + (1/10)

## Discussion

The insertion of foreign sequences, e.g. reporter genes encoding, e.g. enzymes, into a viral genome to generate tagged recombinant viruses, might be possible by inserting the foreign sequence in the place of dispensable parts of the virus [15]. In this study, we report the generation of a recombinant nluc-tagged SBV. The nluc gene was inserted into the region of the Gc-head (N-terminal part of the Gc protein), which was previously described to be dispensable for virus replication in cell culture for Bunyamwera virus, a related virus from the same genus *Orthobunyavirus* [27] and also for SBV [19]. SBV variants lacking parts of the Gc are also described to occur in nature. A hypervariable region was previously identified in the coding region of the Gc-head domain. An increased occurrence of such genetic variations was observed in strains from malformed fetuses [17,18]. We chose the virus variant SBV_D281/12 which carries a natural deletion of 555 nt in the N-terminal part of the Gc [19] and inserted the nluc gene into that locus and rescued the recombinant virus.

The nluc-tagged virus showed the same growth behaviour as the parental virus and replicated to titres as high as the parental virus (10^6^ to 10^7^ TCID_50_/ml). The stability of the nluc was demonstrated throughout eight consecutive passages. No signs of attenuation of the tagged virus were observed in BHK cells. This and the detection of luminescence signals indicated that both the nluc enzyme and the remaining part of the Gc protein could fold correctly, as it was predicted by Alphafold modelling. Therefore, it can be presumed that the nluc tag was successfully integrated without harming *in vitro* virus functions. The nluc-tagged virus showed to be a very sensitive tool to investigate virus replication in cell culture. Every viral particle is thought to have 720 copies of each glycoprotein, as it is described for other members of the *Bunyavirus* family [28]. Besides the glycoproteins with nluc that are free and those from damaged particles, every intact viral particle is thought to carry 720 nluc enzymes, one in each Gc protein. The Gc protein forms trimeric spikes on the surface of the viral particle [29]. Therefore, the nluc enzyme is expressed on the surface of the viral particles in lieu of the missing part of the Gc-head domain. This accounts for a very sensitive detection in the nluc assay. This is a huge advantage over classical methods using non-tagged viruses and allows detailed investigations of viral infection dynamics.

We observed that after only 10 min of virus incubation on cells, a strong luminescence signal was detectable, which did not rise much further after the longer incubation periods of up to 1 h. This demonstrated how rapidly the virus uptake takes place for SBV. The viral glycoproteins Gn and Gc form spikes on the virion surface and are involved in virus attachment [27]. For other *orthobunyaviruses* such as Oropouche virus and La Crosse virus, cell entry via clathrin-mediated endocytosis has been described [30,31]. For SBV and the related Akabane virus, heparan sulphate was demonstrated to be a major entry factor [32]. In our nluc-tagged SBV, a major part of the Gc is missing. However, virus entry still appears to be highly efficient. In accordance with our results for SBV, for the related Bunyamwera virus, virus attachment to the cell surface was also detected as early as 10 min for viruses with a truncated Gc [16]. Therefore, several mechanisms for virus attachment seem likely and should be investigated in more detail in the future.

The onset of viral replication was measured from 6 hpi on in the nluc assay (an increase of luminescence signal of about one log10 level), reaching its highest level at 20 hpi (signal increase of five log10 levels). Similar results are described for an eGFP-tagged Bunyamwera virus, which also carries its tag in place of the N-terminal part of the Gc. An increase in the immunofluorescent signal, which was interpreted as building of progeny viral particles, was likewise observed from 6 hpi on [16]. Therefore, we may conclude that the onset of virus replication is very fast throughout the genus *Orthobunyavirus*.

Despite the 555 nt deletion of the nluc-SBV within the Gc-head, which is the major antigenic domain of SBV [29,33], it was still possible to neutralize the virus by neutralizing antibodies. This shows that the remaining regions were sufficient and that the virus might be a candidate for an SNT. In diagnostics, tagged viruses or tagged pseudoviruses are widely used for investigating neutralizing antibodies in high-throughput assays [34,35]. We, therefore, evaluated our nluc-tagged SBV for the application in an nluc activity-based serum neutralization assay. Conventional protocols are labour-intensive and take multiple days from performing the test to the read-out of the result, while the described nluc-SNT only takes 24 h to perform. In contrast to classic CPE-based protocols, which require experienced personnel for the CPE read-out under a microscope, the nluc-SNT can be evaluated using an automatic luminescence reader. Therefore, this method is more objective than the rather subjective microscopic evaluation of CPE. In the nluc-SNT, the determined neutralization titres of the serum samples from an international proficiency trial for SBV diagnostics were slightly lower than in the conventional SBV SNT. One has to keep in mind that a part (185 aa) of the major immunogenic domain (Gc-head) [29,33] is missing in the nluc-tagged SBV in contrast to the SBV used in the conventional SNT, which usually has an intact Gc. However, not all of the antigenic domains have been deleted, and it appears that enough remains for neutralizing antibodies to bind to, as neutralizing antibodies are directed against several regions of the Gc. The antigenicity of the Gc-deletion mutant with the integrated nluc might be altered and should be explored in the future.

Moreover, the lower titres of the nluc-SNT in comparison to the conventional SNT may be explained by the different calculation method of the titre. The classical SNT titre is indicated as the unit ‘neutralizing doses 50%’ (ND50), and the nluc-SNT titre is indicated as the unit ‘neutralizing doses’. This is the dilution factor, in which all replicates showed neutralization of the virus. Therefore, titres appear lower in this calculation method. This method was chosen because it is the simplest one for automatic read-out. Besides this explanation for the lower titres in the nluc-SNT, one has to keep in mind that the classical SNT also varies in ND50 titre. When looking at the results from the international proficiency trial of SBV diagnostics, where classical SNT was performed by different laboratories all over the world, a broad variation of the ND50 titres was observed [26]. Therefore, the discrimination of positive and negative is more important than the height of the titre.

Other possible areas for the application of the tagged SBV are high-throughput assays for the screening of antiviral reagents. For SBV infections, no effective antiviral treatment is known up to now and screening methods for antiviral agents are also not available. Likewise, for the related human pathogenic Oropouche virus (OROV), no antiviral treatment options are available [36]. In regard to the ongoing OROV outbreak in South America [37], antiviral treatments are urgently needed. For the related orthobunyavirus Bunyamwera virus, an mCherry-tagged virus was successfully applied to screen for antiviral reagents [38]. Similarly, our tagged SBV could be applied for high-throughput screening of antiviral compounds against SBV and could also act as a model for OROV, which can be handled under biosafety level (BSL) 2 instead of BSL 3 conditions.

Overall, this type of tagged virus is a suitable tool that further facilitates the study of viral infection dynamics of orthobunyaviruses and also enables necessary high-throughput assays, which require less labour-intensive or time-consuming procedures.

## Abbreviations

BSL: biosafety level
CPE: cytopathic effect
FCS: fetal calf serum
hpi: hours post-infection
L: large
M: medium
MEM: Minimum Essential Medium
MOI: multiplicity of infection
nluc: nanoluciferase
OROV: Oropouche virus
P: passage
RGS: reverse genetics system
RLU: relative light units
rSBV: recombinant SBV
S: small
SBV: Schmallenberg virus
SNT: serum neutralization test
TCID50/ml: 50% tissue culture infective dose per ml
UTR: untranslated region
